# Image-Guided Radiotherapy for Locally Advanced Head and Neck Cancer

**DOI:** 10.3389/fonc.2013.00172

**Published:** 2013-07-08

**Authors:** Nam P. Nguyen, Sarah Kratz, Claire Lemanski, Jacqueline Vock, Vincent Vinh-Hung, Olena Gorobets, Alexander Chi, Fabio Almeida, Michael Betz, Rihan Khan, Juan Godinez, Ulf Karlsson, Fred Ampil

**Affiliations:** ^1^Department of Radiation Oncology, University of Arizona, Tucson, AZ, USA; ^2^Division of Hematology/Oncology, University of Arizona, Tucson, AZ, USA; ^3^Department of Radiation Oncology, Centre Val d’Aurelle, Montpellier, France; ^4^Department of Radiation Oncology, Lindenhofspital, Bern, Switzerland; ^5^Department of Radiation Oncology, University Hospitals of Geneva, Geneva, Switzerland; ^6^Department of Oral Maxillofacial Surgery, The Ministry and Education of Ukraine, Kiev, Ukraine; ^7^Department of Radiation Oncology, University of West Virginia, Morgantown, WV, USA; ^8^Southwest PET Institute, Tucson, AZ, USA; ^9^Department of Radiation Oncology, Hirslanden Radiation Oncology Institute, Lausanne, Switzerland; ^10^Department of Radiology, University of Arizona, Tucson, AZ, USA; ^11^Florida Radiation Oncology Group, Palatka, FL, USA; ^12^Department of Radiation Oncology, Marshfield Clinic, Marshfield, WI, USA; ^13^Department of Radiation Oncology, Louisiana State University, Shreveport, LA, USA

**Keywords:** head and neck cancer, image-guided radiotherapy, preservation of radiosensitive organs

## Abstract

Treatment of locally advanced head and neck cancer remains a challenge because of the head and neck complex anatomy and the tumor invasion to the adjacent organs and/or metastases to the cervical nodes. Postoperative irradiation or concurrent chemoradiation may lead to damage of radiosensitive structures such as the salivary glands, mandible, cochlea, larynx, and pharyngeal muscles. Xerostomia, osteoradionecrosis, deafness, hoarseness of the voice, dysphagia, and aspiration remain serious complications of head and neck irradiation and impair patient quality of life. Intensity-modulated and image-guided radiotherapy by virtue of steep dose gradient and daily imaging may allow for decreased radiation of the organs at risk for complication while preserving loco-regional control.

## Treatment of Locally Advanced Head and Neck Cancer

Treatment of locally advanced head and neck cancer remains a challenge because of the high rate of loco-regional failures and the potential for serious complications following treatment. The tumor frequently invades adjacent organs and/or regional neck nodes. Standard of care has been either postoperative irradiation or concurrent chemoradiation (1). Regardless of the modality chosen, serious complications may occur because of the presence of radiosensitive organs such as the salivary glands, cochlea, mandible, larynx, and pharyngeal muscles in the radiation field. Xerostomia, deafness, osteoradionecrosis, dysphagia, weight loss, chronic hoarse voice, and aspiration are potential long-term complications of radiation treatment with conventional radiotherapy techniques. Intensity-modulated radiotherapy (IMRT) has been introduced to decrease the toxicity of irradiation because of the steep dose gradient allowing for sparing of radiosensitive organs. Randomized studies have demonstrated significant sparing of the parotid glands following IMRT of head and neck cancer and decreased severity of the xerostomia with improvement of patient quality of life (QOL) (2, 3). However, a significant amount of normal tissues is still irradiated because the inclusion of the tumor and areas at high risk for invasion with a large rim of normal tissue called planning target volume or PTV, to avoid marginal miss. Recently, image-guided radiotherapy (IGRT) by combining the steep dose gradient of IMRT and daily imaging may potentially improve further the toxicity of head and neck irradiation because of the possibility of safe PTV reduction given the reduced inter-fraction movement through daily imaging.

Significant reduction of spinal cord dose may be achieved with IGRT compared to IMRT by a reduced PTV margin (4). However, the flip side of IGRT is also the risk of under-dosing the tumor if the target area is not adequately outlined. Thus, pre-treatment imaging to meticulously delineate the tumor and areas at risk of invasion is a critical component for the success of IGRT.

## Imaging Studies Critical for IGRT Planning

Positron-emission tomography (PET) scan or PET-computed tomography (PET-CT) allows accurate delineation of the tumor and cervical lymph nodes that can be incorporated into the planning CT. PET-CT is superior to CT for tumor imaging because of its ability to detect the tumor metabolic activity in addition to its anatomic location. In a study of 102 unresectable head and neck cancer, PET-CT significantly changed the staging and management of these patients compared to CT alone (5). Twelve patients had modifications of the radiotherapy planning following review of their PET-CT. In another study of 20 patients with oropharyngeal cancers, the incorporation of PET-CT into radiotherapy planning prevented marginal miss in two patients (6). The ability of PET-CT for better tumor delineation compared to CT for radiotherapy planning was also corroborated in other studies (7, 8). Thus, PET-CT should be included in the planning for head and neck IGRT.

Although PET-CT is the diagnostic imaging of choice for head and neck cancer IGRT, magnetic resonance imaging (MRI) also plays a critical role when there is suspicion of nerves infiltration, base of skull or parapharyngeal space invasion by the tumor given its better soft tissue discrimination compared to CT. For patients with nasopharyngeal cancer, MRI is complementary to PET-CT because of the tumor location with high risks for intracranial invasion through the skull base foramen and parapharyngeal extension (9, 10). In addition head and neck MRI may also have a prognostic value for survival after head and neck irradiation of nasopharyngeal cancer (11).

## Image-Guided Radiotherapy Potential for Parotid Gland Preservation

Xerostomia remains one of the most common complications of head and neck cancer Irradiation and may severely affect the QOL of patients. Xerostomia results from apoptosis of the acinar glands secondary to radiation and its severity is proportional to the radiation dose to the parotid glands (12).

Compared to the three-dimensional conformal radiotherapy technique (3D-CRT), IMRT may significantly reduce radiation dose to the parotid glands because of the steep dose gradient. Mean dose to the parotids is usually kept around 26 Gy to allow recovery of the saliva following head and neck irradiation. However, if only one parotid gland can be spared from radiation, current recommendation is to keep mean parotid dose at 20 Gy or lower (13). A recent study suggested that preservation of the contralateral parotid gland may lead to improvement of patient QOL following head and neck cancer irradiation. Among 31 patients with head neck cancer treated with IMRT, there was significant preservation of salivary flow and better QOL as measured by QOL questionnaires if the contralateral parotid gland can be preserved because of less sticky saliva (14). Preliminary experience suggests that IGRT may preserve salivary function and QOL without compromising target coverage. In a study of 76 patients treated with IGRT for head and neck cancer, excellent loco-regional control was obtained as the gross tumor was treated to 70.5 Gy in 2.2–2.3 Gy/fraction while most of the patients were able to preserve a good QOL because of parotid preservation (15). In another study of parotid preservation, IGRT can significantly decrease the mean contralateral parotid gland dose to 14 Gy without compromising target coverage, suggesting that IGRT may further improve patient QOL compared to IMRT (16).

## Potential of IGRT for Hearing Preservation

Hearing loss commonly occurred following concurrent head and neck chemoradiation. A significant proportion of head and neck patients had baseline hearing deficit prior to radiation related to their age. Cisplatin can cause hearing loss which is dose-dependent and may exacerbate the elderly patients hearing deficits. When cisplatin is combined with radiotherapy, the hearing loss may worsen because of the radiosensitization effects of cisplatin on the normal cochlea cells. The threshold for hearing deficit ranges from 10 to 13 Gy when radiotherapy is combined with chemotherapy and affects mainly the high frequency range (>4,000 Hz) (17). However, severe deafness may occur when cochlea radiation dose exceeds 47 Gy because the low frequencies range (<3,000 Hz) is then also affected (18). Deafness is a handicap and may lead to social isolation and poor QOL. In addition, it may affect the patient gainful employment because of the difficulty to communicate at work. Thus, lowering cochlea dose below the threshold for hearing deficit may preserve the patients’ hearing and conserve their QOL. Compared to 3D-CRT, IMRT may decrease radiotherapy dose to the cochlea and provide better hearing preservation (19). Mean cochlea dose reported in the literature for patients with head and neck cancer undergoing IMRT ranged from 16 to 55 Gy. Preliminary data for IGRT of head and neck cancer for cochlea sparing is encouraging. In a study of 52 patients who had IGRT for locally advanced head and neck cancer, mean cochlea dose was reduced to 6–6.5 Gy which is below the threshold for radiotherapy damage without compromising target volume coverage (20). Thus, hearing preservation with IGRT is feasible and needs to be investigated in future prospective studies of head and neck cancer.

## Potential of IGRT for Mandibular Sparing

Osteoradionecrosis remains one of the most feared complications of head and neck cancer irradiation because of its effect on patient QOL. In severe cases of osteoradionecrosis unresponsive to conservative management, resection of the damaged bone may result in severe alteration of speech, chewing, and swallowing. The risk of radionecrosis is related to the volume of normal bone radiated to high radiation dose. Damage of the microvasculature irrigating the mandible may lead to decreased blood flow, poor wound healing, and ultimately necrosis. Mandibular radionecrosis usually occurs when the mandible dose exceeds 66 Gy (21). The prevalence of osteoradionecrosis ranges from 5 to 7% in head and neck cancer patients treated with the conventional fractionation (1.8–2 Gy/fraction) and 3D-CRT. The risk of radionecrosis may be reduced with IMRT because of the sharp dose gradient allowing for reduction of the volume of normal bone radiated to a high dose. The reported prevalence of osteoradionecrosis ranges from 1 to 5% depending on the anatomic site of the cancer as cancers of the oral cavity usually require treating a large of volume of the mandible to a high radiation dose (22, 23). The IGRT technique may further decrease radiation dose to the mandible and thus the risk of radionecrosis. In a study of 83 head and neck cancer patients of various anatomic sites treated with IMRT (17) and IGRT (66), only one patient developed radionecrosis (24). Thus, IGRT may be a promising technique for mandibular preservation in future clinical trials.

## Potential of IGRT to Prevent Laryngeal Edema in Non-Laryngeal and Non-Hypopharyngeal Head and Neck Cancer

Laryngeal edema and resulting dysphonia commonly occur following head and neck cancer radiation when the laryngeal dose exceeds 43.5 Gy (25). The dysphonia severity is proportional to the radiation dose and related on the abnormal cord vibrations caused by the edema which may be persistent up to 72 months after radiation (26). Chronic hoarseness of the voice may impair patient QOL or affect their professional activity if the patient depends on the quality of his voice to make a living. Laryngeal edema is unavoidable when the tumor is located in the larynx or hypopharynx because of the high radiation dose required for cure (70 Gy). When the tumor is located in other anatomic sites, shielding of the larynx with a midline block would significantly decrease radiation dose to the larynx and prevent the risk of laryngeal edema. However, in the presence of cervical lymph nodes, a laryngeal shield may under-dose the cervical lymph nodes leading to an increased risk of regional recurrence. Whole field head and neck IMRT is usually recommended in the presence of neck node metastases to avoid geographic miss. Compared to whole field head and neck IMRT, IGRT can significantly reduce radiation dose to the larynx in patients with non-laryngeal and non-hypopharyngeal head and neck cancers (27). Preliminary results suggest that IGRT may also reduce the risk of laryngeal edema and effectively preserve patient voice following head and neck radiation (28).

## Potential of IGRT to Reduce Aspiration Risk Following Radiation for Non-Laryngeal and Non-Hypopharyngeal Head and Neck Cancer

Aspiration is a life-threatening complication of a head and cancer irradiation because of the risk of pneumonia and sepsis. Aspiration commonly occurs following head and neck cancer irradiation because of damage to the pharyngeal constrictor muscles. Aspiration risk is proportional to radiation dose to the pharyngeal muscles. Even though there are still controversies about which pharyngeal muscles are critical for the development of aspiration, 32% of the patients developed aspiration when the dose to the inferior pharyngeal muscles exceeds 52 Gy (29). Thus, patients with laryngeal and hypopharyngeal cancer are at highest risk of aspiration following head and neck cancer irradiation. However, a high rate of aspiration is still observed following radiation of non-laryngeal and non-hypopharyngeal head and neck cancers. Aspiration rates ranged from 16 to 54% following non-laryngeal and non-hypopharyngeal cancer irradiation with 3D-CRT and whole field IMRT (30, 31). Whole field IGRT reduces significantly the risk of aspiration for these patients. Only 2 out of 48 patients developed minimal aspiration which resolved with swallowing therapy following IGRT for non-laryngeal and non-hypopharyngeal head and neck cancer (32). Thus, IGRT is a promising technique to reduce the risk of aspiration and to improve patient QOL in head and neck cancer patients.

## Effectiveness of IGRT for Loco-Regional Control in Patients with Head and Neck Cancers

Preliminary clinical experiences of IGRT for head and neck cancers have been very encouraging. Excellent loco-regional control and survival have been achieved for various anatomic head and neck cancer sites. In a study of 19 patients with locally advanced oral cavity cancer who received postoperative irradiation with IGRT, the 2-year survival and loco-regional control were 94 and 92%, respectively (33). In another study of 28 patients who underwent concurrent chemotherapy and IGRT for locally advanced nasopharyngeal cancer, the 3-year survival and loco-regional control were respectively 83.5 and 88.4% respectively (34). Similar high rates of loco-regional control were also observed for oropharyngeal and laryngeal cancers (35, 36). Because of the small number of patients and the short follow-up, these studies should be interpreted with caution but they may be helpful for the design of future prospective studies of head and neck cancer IGRT.

## Conclusion

Image-guided radiotherapy for head and neck cancer is a promising technique of radiation because of the potential for normal organ sparing without compromise of target coverage. Preliminary clinical results suggest that the dosimetric advantages of IGRT may be translated into excellent loco-regional control for patients with head and neck cancer. Further prospective studies with a large number of patients should be performed to verify this hypothesis.

## Conflict of Interest Statement

The authors declare that the research was conducted in the absence of any commercial or financial relationships that could be construed as a potential conflict of interest.

## References

[1] Soo KC Tan EH Wee J Lim D Tai BC Khoo ML Surgery and adjuvant radiotherapy vs concurrent chemoradiotherapy in stage III/IV non-metastatic squamous cell head and neck cancer. Br J Cancer (2005) 93:279–86 10.1038/sj.bjc.6602696 16012523 PMC2361563

[2] Gupta T Agarwall J Jain S Phurailatpam R Kannan S Ghosh-Laskar S Three-dimensional conformal radiotherapy (3D-CRT) versus intensity modulated radiotherapy (IMRT) in squamous cell carcinoma of the head and neck: a randomized controlled trial. Radiother Oncol (2012) 104:343–8 10.1016/j.radonc.2012.07.001 22853852

[3] Nutting CM Morden JP Harrington KJ Urbano TG Bhide SA Clark C Parotid-sparing intensity modulated versus conventional radiotherapy in head and neck cancer (PASSPORT): a phase 3 multicentre randomized controlled trial. Lancet Oncol (2011) 12:127–36 10.1016/S1470-2045(10)70290-4 21236730 PMC3033533

[4] Schwarz M Giske K Stoll A Nill S Huber PE Debus J IGRT versus non-IGRT for postoperative head and neck IMRT patients: dosimetric consequences arising from a PTV reduction. Radiat Oncol (2012) 7:133 10.1186/1748-717X-7-133 22873744 PMC3484069

[5] Abramyuk A Appold S Zophel K Baumann M Abolmaali N Modification of staging and treatment of head and neck cancer by FDG-PET/CT prior to radiotherapy. Strahlenther Onkol (2013) 189:197–201 10.1007/s00066-012-0283-0 23329277

[6] Chatterjee S Frew J Mott J McCallum H Stevenson P Maxwell R Variation in radiotherapy target volume definition, dose to organs at risk and clinical target volumes using anatomic (computed tomography) versus combined anatomic and molecular imaging (positron emission tomography/computed tomography): intensity-modulated radiotherapy delivered using a tomotherapy Hi-Art machine: final results of the VortigERN study. Clin Oncol (2012) 24:e173–9 10.1016/j.clon.2012.09.004 23079100

[7] Delouya G Igidbashian L Houle A Belair M Boucher L Cohade C ^18^F-FDG-PET imaging in radiotherapy volume delineation in treatment of head and neck cancer. Radiother Oncol (2011) 101:362–8 10.1016/j.radonc.2011.07.025 21885143

[8] Garg MK Glantzman J Kalnicki S The evolving role of positron emission tomography-computed tomography in organ-preserving treatment of head and neck cancer. Semin Nucl Med (2012) 42:320–7 10.1053/j.semnuclmed.2012.04.005 22840597

[9] Liao XB Mao YP Liu LZ Tang LL Sun Y Wang Y How does magnetic resonance imaging influence staging according to AJCC staging system for nasopharyngeal carcinoma compared with computed tomography. Int J Radiat Oncol Biol Phys (2008) 72:1368–77 10.1016/j.ijrobp.2008.03.017 18455329

[10] Nemzek WR Hecht S Gandour-Edwards R Donald P Perineural spread of head and neck tumors: how accurate is MR imaging? AJNR Am J Neuroradiol (1998) 19:701–6 9576658 PMC8337413

[11] Tang LL Li WF Chen L Sun Y Chen Y Liu LZ Prognostic values and staging categories of anatomic masticator space involvement in nasopharyngeal carcinoma: a study of 924 cases with MRI imaging. Radiology (2010) 257:151–7 10.1148/radiol.10100033 20697119

[12] Roesink JM Moerland MA Batterman JJ Hordijk G Terhaard CHJ Quantitative dose-volume response analysis of changes in parotid gland function after radiotherapy in the head and neck region. Int J Radiat Oncol Biol Phys (2001) 4:938–46 10.1016/S0360-3016(01)01717-5 11704314

[13] Deasy JO Moisenko V Marks L Chao KSC Nam J Eisbruch A Radiotherapy dose-volume effects on salivary gland function. Int J Radiat Oncol Biol Phys (2010) 76:S58–63 10.1016/j.ijrobp.2009.06.090 20171519 PMC4041494

[14] Chen WC Lai CH Lee TF Hung CH Liu KC Tsai MF Scintigraphic assessment of salivary function after intensity-modulated radiotherapy for head and neck cancer: correlation with parotid dose and quality of life. Oral Oncol (2013) 49:42–8 10.1016/j.oraloncology.2012.07.004 22854066

[15] Voordeckers M Farrag A Everaert H Tournel K Storme G Verellen D Parotid sparing with helical tomotherapy in head and and neck cancer. Int J Radiat Oncol Biol Phys (2012) 84:443–448 10.1016/j.ijrobp.2011.11.070 22836056

[16] Nguyen NP Vos P Vinh-Hung V Ceizyk M Smith-Raymond L Stevie M Feasibility of image-guided radiotherapy based on helical tomotherapy to reduce contralateral parotid dose in head and neck cancer. BMC Cancer (2012) 12:175 10.1186/1471-2407-12-175 22578076 PMC3411401

[17] Hitchcock YJ Tward JD Szabo A Bentz BG Shrieve DC Relative contributions of radiation and cisplatin-based chemotherapy to sensori-neural hearing loss in head and neck cancer patients. Int J Radiat Oncol Biol Phys (2009) 73:779–88 10.1016/j.ijrobp.2008.05.040 18707819

[18] Chan SH Ng WT Kam KL Lee MC Choi CW Yau TK Sensorineural hearing loss after treatment of nasopharyngeal carcinoma: a longitudinal analysis. Int J Radiat Oncol Biol Phys (2009) 73:1335–42 10.1016/j.ijrobp.2008.07.034 18922648

[19] Petsuksiri J Sermsree A Thephamongkhol K Keskool P Thongvai K Chansilpa Y Sensorineural hearing loss after concurrent chemoradiotherapy for head and neck cancer. Radiat Oncol (2011) 6:19 10.1186/1748-717X-6-19 21333025 PMC3048471

[20] Nguyen NP Smith-Raymond L Vinh-Hung V Sloan D Davis R Vos P Feasibility of tomotherapy to spare the cochlea from excessive radiation in head and neck cancer. Oral Oncol (2011) 47:414–9 10.1016/j.oraloncology.2011.03.011 21474364

[21] Glanzmann C Grätz KW Radionecrosis of the mandible: a retrospective analysis of the incidence and risk factors. Radiother Oncol (1995) 36:94–100 10.1016/0167-8140(95)01583-3 7501817

[22] Gomez DR Zhung JE Gomez J Chan K Wu AJ Wolden SL Intensity-modulated radiotherapy in postoperative treatment of oral cavity cancer. Laryngoscope (2009) 73:1096–103 10.1016/j.ijrobp.2008.05.024 18707827

[23] Huang K Xia P Chuang C Weinberg V Glastonbury CM Eisele DW Intensity-modulated chemoradiation for treatment of stage III and IV oropharyngeal carcinoma: the University of California-San Francisco experience. Cancer (2008) 113:497–507 10.1002/cncr.23578 18521908

[24] Nguyen NP Vock C Chi A Ewell L Vos P Mills M Effectiveness of intensity-modulated and image-guided radiotherapy to spare the mandible from excessive radiation. Oral Oncol (2012) 48:653–7 10.1016/j.oraloncology.2012.01.016 22341305

[25] Sanguineti G Adapala P Endres EJ Brack C Fiorino C Sormani P Dosimetric predictor of laryngeal edema. Int J Radiat Oncol Biol Phys (2007) 68:741–9 10.1016/j.ijrobp.2007.01.010 17398024

[26] Hamdan A Geara F Rameh C Husseini HT Eid T Fuleihan N Vocal changes following radiotherapy to the head and neck for non-laryngeal tumors. Eur Arch Otorhinolaryngol (2009) 266:1435–9 10.1007/s00405-009-0950-7 19319555

[27] Nguyen NP Ceizyk M Vos P Vinh-Hung V Davis R Desai A Effectiveness of image-guided radiotherapy for laryngeal sparing in head and neck cancer. Oral Oncol (2010) 46:283–6 10.1016/j.oraloncology.2010.01.010 20188620

[28] Nguyen NP Abraham D Desai A Betz M Davis R Sroka T Impact of image-guided radiotherapy to reduce laryngeal edema following treatment for non-laryngeal and non-hypopharyngeal head and neck cancer. Oral Oncol (2011) 47:900–4 10.1016/j.oraloncology.2011.06.004 21724448

[29] Caglar HB Tishler RB Othus M Burke E Li Y Goguen L Dose to larynx predicts for swallowing complications after intensity-modulated radiotherapy. Int J Radiat Oncol Biol Phys (2008) 72:1110–8 18468812 10.1016/j.ijrobp.2008.02.048

[30] Feng FY Kim HM Lyden TH Haxer MJ Worden FP Feng FM Intensity-modulated chemoradiotherapy aiming to reduce dysphagia in patients with oropharyngeal cancers. J Clin Oncol (2010) 28:2732–8 10.1200/JCO.2009.24.6199 20421546 PMC2881852

[31] Nguyen NP Frank C Moltz CC Vos P Smith HJ Nguyen PD Analysis of factors influencing aspiration rates following chemoradiation for head and neck cancer. Br J Radiol (2009) 82:675–80 10.1259/bjr/72852974 19332514

[32] Nguyen NP Smith-Raymond L Vinh-Hung V Vos P Davis R Desai A Feasibility of Tomotherapy based image-guided radiotherapy to reduce aspiration risk in patients with non-laryngeal and non-hypopharyngeal head and neck cancer. PLoS One (2013) 8:e56290 23505414 10.1371/journal.pone.0056290 PMC3591427

[33] Hsieh C Kuo Y Liao L Hu K Lin S Lin Y Image-guided intensity-modulated radiotherapy with helical tomotherapy for postoperative high risk oral cavity cancer. BMC Cancer (2011) 11:37 10.1186/1471-2407-11-37 21269518 PMC3037924

[34] Shueng PW Shen BJ Wu LJ Liao LJ Hsiao CH Lin YC Concurrent image-guided guided intensity modulated radiotherapy and chemotherapy following neoadjuvant chemotherapy for locally advanced nasopharyngeal carcinoma. Radiat Oncol (2011) 6:95 10.1186/1748-717X-6-95 21838917 PMC3163198

[35] Nguyen NP Ceizyk M Vos P Betz M Chi A Almeida F Feasibility of tomotherapy-based image-guided radiotherapy for locally advanced oropharyngeal cancers. PLoS ONE (2013) 8:e60268 10.1371/journal.pone.0060268 23555938 PMC3610680

[36] Nguyen NP Chi A Betz M Almeida F Vos P Davis R Feasibility of intensity-modulated and image-guided radiotherapy for functional organ preservation in locally advanced laryngeal cancer. PLoS ONE (2012) 7:e42729 10.1371/journal.pone.0042729 22916151 PMC3423414

